# Multifunctional Cellulose and Cellulose-Based (Nano) Composite Adsorbents

**DOI:** 10.3389/fbioe.2022.891034

**Published:** 2022-04-14

**Authors:** Ru-Jie Shi, Tian Wang, Jia-Qi Lang, Nong Zhou, Ming-Guo Ma

**Affiliations:** ^1^ Chongqing Engineering Laboratory of Green Planting and Deep Processing of Famous-Region Drug in the Three Gorges Reservoir Region, College of Biology and Food Engineering, Chongqing Three Gorges University, Chongqing, China; ^2^ Engineering Research Center of Forestry Biomass Materials and Bioenergy, Beijing Key Laboratory of Lignocellulosic Chemistry, Research Center of Biomass Clean Utilization, College of Materials Science and Technology, Beijing Forestry University, Beijing, China

**Keywords:** adsorbents, cellulose, (nano) composites, heavy metals ions, organic pollutants

## Abstract

In recent years, faced with the improvement of environmental quality problems, cellulose and cellulose-based (nano) composites have attracted great attention as adsorbents. In this review article, we first report the recent progress of modification and functionalization of cellulose adsorbents. In addition, the adsorbents produced by the modification and functionalization of carboxymehyl cellulose are also introduced. Moreover, the cellulose-based (nano) composites as adsorbents are reviewed in detail. Finally, the development prospect of cellulose and cellulose-based (nano) composites is studied in the field of the environment. In this review article, a critical comment is given based on our knowledge. It is believed that these biomass adsorbents will play an increasingly important role in the field of the environment.

## Introduction

In recent years, many environmental problems have attracted more and more attention because they are related to human survival and development (Jarup and Akesson, 2009). Heavy metals ions and organic pollutants play an important role in human health, disturbing the normal growth of plants and damaging the ecological balance (Heppner et al., 2009). Some heavy metals ions and organic pollutants are teratogenic, mutagenic, and carcinogenic substances. There is no doubt that heavy metals ions and organic pollutants accumulate in the environment and biological energy is enriched through the food chain. Therefore, it is very important to remove these pollutants using adsorbents from wastewater (Chen et al., 2015).

It is believed that lignocellulose is an important renewable biomass, consisting of cellulose, hemicelluloses, and lignin, which is used as feedstock to fabricate bio-based fuels, chemicals, and materials (Menon and Rao, 2012; Huang et al., 2018; Dong H. et al., 2020; Huang et al., 2021b). Cellulose is a polysaccharide composed of glucose molecules and the most abundant renewable biomass in the world. Cellulose is the main component of the plant cell wall. It is expected that the conversion of bio-ethanol from cellulose will be a clean energy technology, replacing the traditional grain ethanol technology to meet global energy demand (Asgher et al., 2013). However, it is still difficult to achieve large-scale industrialization due to the pretreatment of raw materials and the high cost of cellulose (Mansfield et al., 1999). Therefore, bio-based chemicals and materials produced from cellulose are expected to be beneficial to the application of cellulose (Nzediegwu and Dumont, 2021). It is believed that bio-based materials produced from cellulose have become the main research focus of academia (Klemm et al., 2005; Li et al., 2011; Liu et al., 2017; Cao et al., 2018). In addition, carboxymehyl cellulose is an organic substance, a carboxymethylated derivative of cellulose, which is easy to disperse in water to form a transparent colloidal solution.

It is well known that adsorption is considered to be an economic route to remove heavy metals ions and organic pollutants from wastewater (Yagub et al., 2014). In addition, the adsorbent could be reused through an appropriate desorption and regeneration process. Activated carbon adsorbents are widely used to remove heavy metal pollutants due to their large pore volume and high surface area (Mohan and Pittman, 2006; Liu et al., 2018; Liu et al., 2019). To date, hundreds of adsorbents have been reported in the literature (Babel and Kurniawan, 2003; Crini, 2006). The cellulose and cellulose-based (nano) composites have different compositions, structures, and properties, compared with activated carbon adsorbent. It is believed that bio-sorption of agricultural wastes, by-products, and natural substances is a promising and emerging adsorbent method for the treatment of heavy metals ions and organic pollutants due to its high efficiency and wide sources (Volesky and Holan, 1995; O'Connell et al., 2008).

Herein, this review article introduces the application of cellulose and cellulose-based (nano) composites in the removal of heavy metals ions and organic pollutants from wastewater. The adsorbents produced by modification and functionalization of cellulose, carboxymehyl cellulose, and cellulose-based (nano) composites are reviewed. In addition, the adsorption mechanism is briefly discussed. Finally, we try to put forward the possible future development of cellulose and cellulose-based materials in the field of the environment.

## Adsorbents Produced by Modification and Functionalization of Cellulose

Heavy metals ions and organic pollutants are the main factors causing wastewater pollution. The modification and functionalization of cellulose are usually utilized to create adsorbents to remove heavy metals ions. In early 2001, Liu’s group did pioneering work (Liu et al., 2001). They developed spherical cellulose as an adsorbent to remove and recover Cr^3+^ with a recovery rate of approximately 85.2%, following the predominant complex adsorption mechanism. It is reported that the adsorption of Cr^3+^ ions by an adsorbent depends on time, concentration, pH, and temperature. Then, Xu et al. (2002) also reported the removal of Cd^2+^ by immobilized cellulose-binding domains synthetic phytochelatin bio-adsorbents at the level of per million. Shukla and Pai (2005) assessed the cellulose-containing biomass for Pb^2+^ removal. The results showed the maximum cation uptake value of 0.127 mmol g^−1^ for coir, 0.087 mmol g^−1^ for sawdust, 0.090 mmol g^−1^ for jute, and 0.106 mmol g^−1^ for groundnut shell. Acrylonitrile-grafted cyanoethyl cellulose was also formed from cyanoethyl cellulose by the ionic-xanthate method to graft the acrylonitrile (Kamel et al., 2006). Undoubtedly, these early works showed that the modification and functionalization of cellulose is a promising bio-adsorbent method to remove heavy metals ions. After that, more and more research groups have paid more attention to the synthesis, properties, and application potential of bio-adsorbents.

O'Connell et al. (2006b) produced a regenerated cellulose adsorbent. It was found that glycidyl methacrylate-modified cellulose could remove 72 mg g^−1^ of Pb^2+^ ions from aqueous solution at 23°C. In addition, the adsorbent was also applied to remove Ni^2+^ ions from an aqueous solution with approximately 48 mg g^−1^ removed (O'Connell et al., 2006a). It should be pointed out, however, that there are still some debates about the function of cellulose. Garcia-Reyes and Rangel-Mendez (2009) demonstrated that hemicellulose and lignin were the main contributors for the removal of Cr^3+^ in aqueous solution, while cellulose did not seem to be involved. Recently, a microcrystalline cellulose (MCC) adsorbent was used to remove Pb^2+^ ions from aqueous solution with 1,2,3,4-butanetetracarboxylic acid (Hashem et al., 2020). According to the Langmuir theory, the maximum adsorption capacity was 1,155 mg g^−1^ at pH 5 and 30°C. The pseudo-second order kinetic model indicated the chemisorption in the adsorption process due to the high density of active sites. It observed the endothermic and nonspontaneous adsorption of Pb^2+^ ions by MCC.

Cadmium, a typical heavy metal, has the characteristics of high fat solubility, bioaccumulation, and toxicity, contributing to wastewater pollution. Belhalfaoui and co-workers chemically modified cellulose with succinic anhydride in toluene to obtain succinylated cellulose (Belhalfaoui et al., 2009). Sodium succinylated cellulose displayed high sorption efficiency and high selectivity to remove Cd^2+^ with a maximum uptake of 185.2 mg g^−1^ for distilled water and 178.6 mg g^−1^ for spiked groundwater solutions. It suggested that active functional groups played a major role in metal sorption. It followed an ion-exchange mechanism in the removal process. We would like to point out that this work provided a very valuable example for the research of adsorption mechanisms. In Lu’s work, the different adsorption capacities from Langmuir-Freundlich were carried out using lawny grass modified by citric acid as an adsorbent to remove Cd^2+^ from aqueous solution (Lu et al., 2010).

**FIGURE 1:**
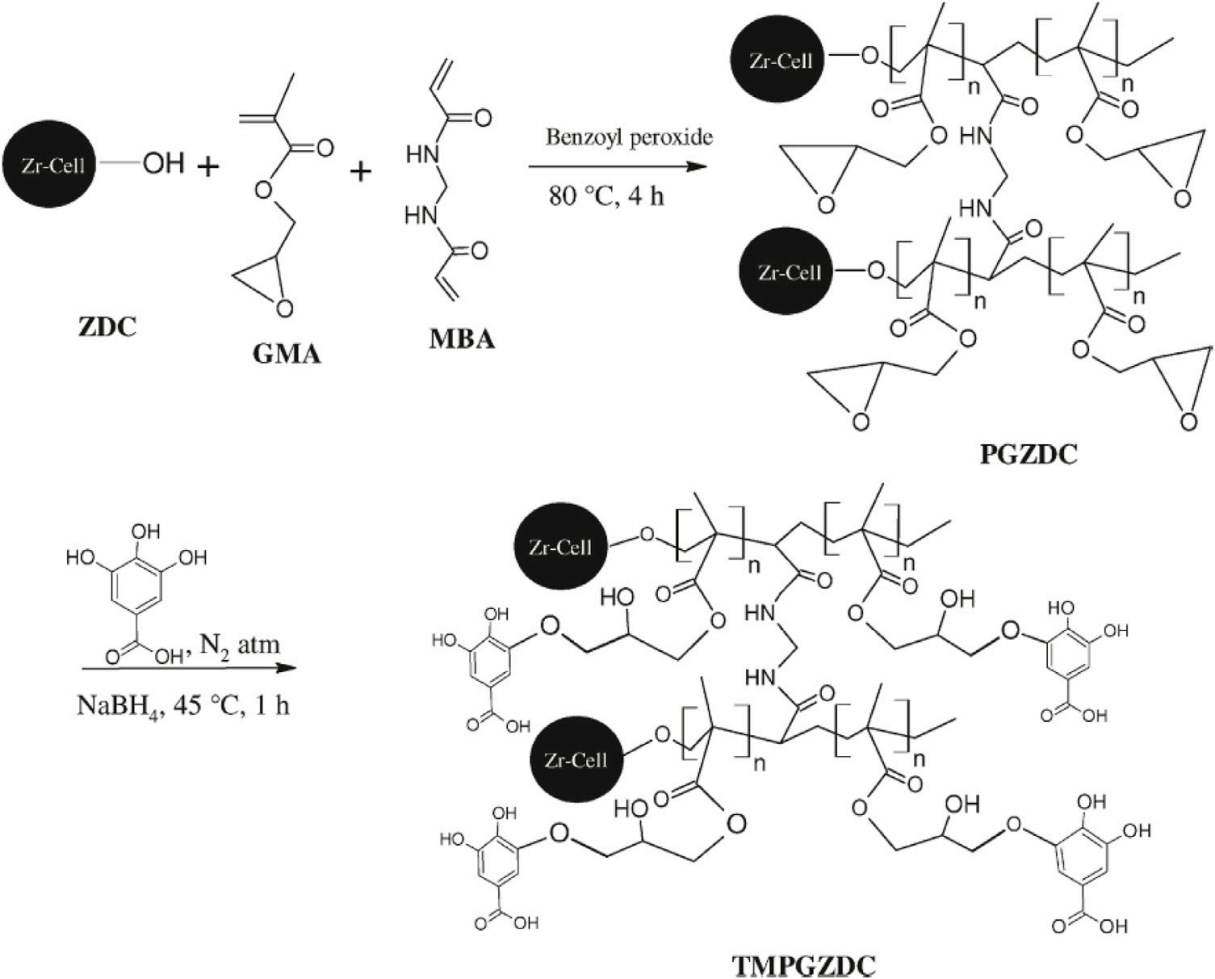
Proposed reaction mechanism for the synthesis of tannin-modified poly (glycidylmethacrylate)-grafted zirconium oxide densified cellulose (TMPGZDC): zirconium oxide densified cellulose (ZDC) grafted with glycidylmethacrylate (GMA), N,N0-methylenebisacrylamide (MBA), and poly (glycidylmethacrylate)-grafted zirconium oxide densified cellulose (PGZDC) (Anirudhan and Rejeena, 2011).

In the literature, the modification and functionalization of cellulose were also applied to absorb toxic and radioactive elements such as Hg^2+^, UO_2_^2+^, Th^4+^, and arsenate anions. It also reported that 95.5% alpha-cellulose grafting with acrylamide was utilized as an adsorbent to remove Hg^2+^ ions with an adsorption capacity of 625 mg g^−1^ (Hashem et al., 2006). Takagai et al. (2011) investigated different thio-modified cellulose resins to remove Hg^2+^ ions in acidic solutions. Furthermore, three silylcellulosic derivatives of cellulose, trimethylsilyl-cellulose, and triphenylsilyl-cellulose with different substitution degrees were examined as sorbents for uranyl ions in wastewaters. The results revealed that the complexation of cellulose and trimethylsilyl-cellulose with UO_2_^2+^ improved the thermal stability (Bontea et al., 2006). Anirudhan and Rejeena (2011) synthesized a tannin-modified poly (glycidylmethacrylate)- grafted zirconium oxide-densified cellulose adsorbent. The procedure adopted for the preparation of the adsorbent is presented in Figure 1. The authors indicated that the pH was found to be 5.5 with the adsorption rate of 99.2% for Th^4+^. Moreover, an Fe^3+^-loaded ligand exchange cotton cellulose macroporous bead adsorbent was applied for selective adsorption of arsenate anions by Zhao et al. (2009). The cellulose nanofibrils (CNFs) were reported to have various applications (Cao et al., 2019; Cao et al., 2020; Huang et al., 2020; Wang P. et al., 2020). More recently, CNFs were used to adsorb Hg^2+^ ions in an aqueous solution (Bisla et al., 2020). Modified CNFs had an adsorption capacity of 131.86 mg g^−1^ for Hg^2+^ ions. The pseudo-second order kinetic model implied chemisorption during the removal of Hg^2+^ ions from simulated wastewater. The diethylenetriaminepentaacetic acid-modified cellulose was obtained by a pre-grafting technique (Li et al., 2021). The adsorbent showed an ultrahigh adsorption capacity of 443.8 mg g^−1^ for Hg^2+^ in aqueous solution. A total of 88.13% of the original adsorption capacity was maintained after five cycles of the regeneration process. Aminotriazole isomer-modified cellulose microspheres with high nitrogen content showed potential affinity for U^6+^ (Wen et al., 2021). The microspheres possessed good adsorption capacity, selectivity, and reusability for U^6+^. It removed 99.45% from uranium contaminated water, 21.68% from contaminated groundwater, and 75.97% from simulated seawater.

Generally, the modification and functionalization of cellulose can remove all kinds of heavy metals ions simultaneously. In fact, wastewater contains a variety of heavy metals ions. There are some reports about this type of research. For example, Guclu et al. (2003) found four types of cellulose graft copolymers that removed Pb^2+^, Cu^2+^, and Cd^2+^ ions from aqueous solution. Cellulosic materials containing grafted polyacrylonitrile and poly (acrylic acid) molecules were also reported to remove Cd^2+^ and Cu^2+^ ions from aqueous solutions (Okieimen et al., 2005). Norkus et al. (2006) applied an oxygen delignified alkaline cellulose pulp slurry to remove Fe, Mn, and Cu. The maximum adsorption capacities of Ni^2+^, Co^2+^, Zn^2+^, and Cd^2+^ were 1.28, 1.23, 1.21, and 1.13 mol kg^−1^ by using chemically modified orange peel cellulose adsorbents, which increased by 95, 178, 60, and 130%, respectively, compared with that of raw orange peel (Li et al., 2008). The modified sugarcane bagasse and chemically modified cellulose displayed adsorption capacities for Ca^2+^ from 15.6 to 54.1 mg g^−1^ and Mg^2+^ from 13.5 to 42.6 mg g^−1^ (Karnitz et al., 2010). Recently, modified cellulose hydrogels were reported for the adsorption of heavy metals ions by the ion-exchange mechanism (Zhao et al., 2019). The maximum absorption capacity of 157.51, 393.28, and 289.97 mg g^−1^ for Cu^2+^, Pb^2+^, and Cd^2+^ ions was found in modified cellulose hydrogels, respectively. The thiol-functionalized cellulose nanofiber membrane was reported to adsorb heavy metals ions by chemisorption (Choi et al., 2020). It achieved adsorption capacities of 49.0 mg g^−1^ for Cu^2+^, 45.9 mg g^−1^ for Cd^2+^, and 22.0 mg g^−1^ for Pb^2+^ in the Langmuir isotherm. The microwave-functionalized cellulose derived from rice husk was reported to eliminate Pb^2+^, Cd^2+^, and Ni^2+^ (Qu et al., 2020). It achieved adsorption capacities of 295.20 mg g^−1^ for Pb^2+^, 151.51 mg g^−1^ for Cd^2+^, and 72.80 mg g^−1^ for Ni^2+^. The functionalized cellulose was found to have good recoverability and adsorption efficiency after five cycles.

Besides the removal of heavy metals ions, the modification and functionalization of cellulose are also applied to the removal of inorganic pollutants. Inukai et al. (2004) firstly used *N*-methylglucamine-type cellulose derivatives to remove B^3+^. Then, Fe^3+^-loaded ligand exchange cotton cellulose as a bead adsorbent was reported to remove fluoride from drinking water by Zhao et al. (2008). Anirudhan et al. (2009) synthesized a cellulose-based anion exchanger bearing the –N^+^HR_2_Cl^−^ functional group with the adsorption capacity of 197.75 mg g^−1^ for V^5+^ at 30°C. After that, they demonstrated that about 99.6% of phosphate was adsorbed in 180 min of contact at 100 mg L^−1^ by using a cellulose-grafted epichlorohydrin functionalized polyethylenimine graft copolymer as an adsorbent (Anirudhan et al., 2012). Besides the removal of heavy metals ions, it is also important for the modification and functionalization of cellulose to remove organic pollutants. For example, Fang et al. (2004) prepared a lysine-cellulose bead adsorbent for removing bacterial endotoxins. The adsorbent had the characteristics of mechanical strength, blood compatibility, and cytotoxicity. Alila and Boufi (2009) constructed a modified cellulose fiber adsorbent to remove several aromatic organic compounds and three herbicides of Alachlor, Linuron, and Atrazine. More recently, a cellulose acetate fiber membrane was reported to remove methylene blue (MB) and Congo red (CR) dyes (Chen et al., 2020). It was observed that the adsorption capacities were 69.89 mg g^−1^ for MB and 67.31 mg g^−1^ for CR. An anionic cellulose foam was obtained by grafting and chemical crosslinking (Feng et al., 2020). It exhibited an adsorption capacity of 364.22 mg g^−1^ for anionic dye Eosin Y and a removal efficiency of 99.58%. The chemical and monolayer action anionic dyes were suggested during the adsorption procedure.

There have been many reports about the removal of lipoprotein by modification and functionalization of cellulose. As early as 1988, Franceschini et al. (1988) comparatively tested a dextran sulfate cellulose column and double membrane filtration for the extracorporeal removal of low density lipoproteins. Schulzeck et al. (1992) further discovered five patients with familial hypercholesterolemia and diet- and drug-resistant low-density lipoprotein cholesterol greater than 230 mg dl^−1^ using dextran sulfate cellulose adsorption. Olbricht (1996) indicated the extracorporeal removal of low-density lipoprotein cholesterol by dextran sulfate cellulose adsorption. It reported that the reduction in cholesterol per treatment was 65–75% and in most patients one treatment per week was sufficient to reduce cholesterol to 100–150 mg dl^−1^. In Wang’s work, a cellulose adsorbent with amphiphilic ligands was applied to adsorb low-density lipoprotein with a better selectivity and adsorption capacity for the removal of low density lipoprotein (LDL), total cholesterol (TC), total proteins (TP) at 0.857, 1.317, and 1.002 mg ml^−1^, respectively (Wang et al., 2002). The adsorbent showed quite good adsorption performance for selective removal of LDL from human plasma (Yu et al., 2006). Wang et al. (2006) synthesized a carboxyl modified polyethylene glycol (PEG) spacer and linked it covalently to cellulose beads. Both the adsorption capacity and adsorption efficiency of the ligand was increased for adsorption of LDL-cholesterol and the average adsorption capacity of LDL-cholesterol was increased to 0.903 mg ml^−1^.

Beutler and Gelbart (1986) reported that cellulose columns efficiently removed leukocytes from whole blood, and the leukocyte removal activity of cellulose columns was due to mechanical filtration. Weber et al. (1995) produced regenerated cellulose (RC) of 1–8 μm modified with polyethyleneimine or diethylaminoethyl groups. RC had high adsorption capacity for endotoxins in human plasma. Butnaru et al. (2003) discussed the treatment of wastewater from cellulose dyeing with direct dyes by electro-coagulation. A cellulose microporous hollow fiber membrane was applied for the dispersion-free reactive extraction of thiol compounds (Yang et al., 2007). Greben et al. (2007) demonstrated that salinity (sulphate) could be removed using the fermentation products of grass-cellulose. The polycation-immobilized pore cellulose spherical particles were also reported to remove the endotoxin by Sakata et al. (2007).

**FIGURE 2:**
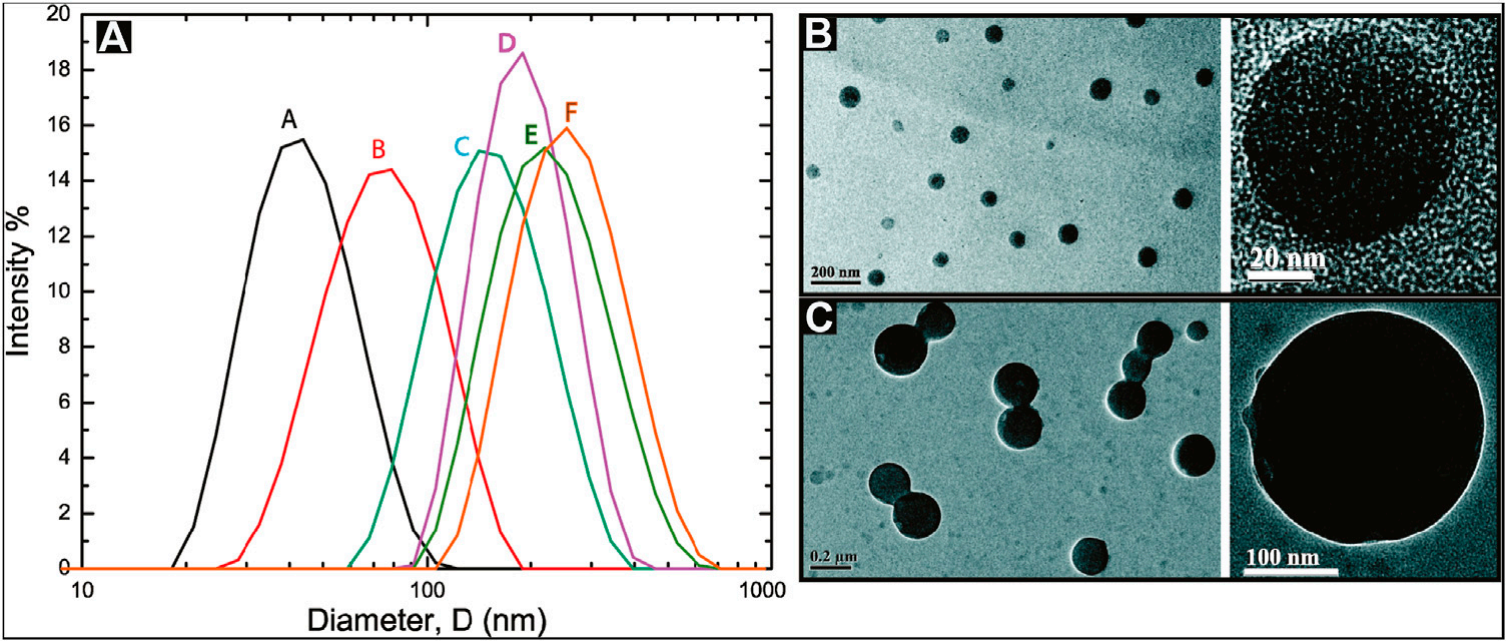
**(A)** Intensity-weighted particle size distributions for polymeric nanoparticulate drug delivery systems (PNDDS). The mean values of Z-average sizes of different batches of PNDDS were 46 ± 1 nm (black, A), 81 ± 2 nm (red, B), 159 ± 1 nm (cyan, C), 197 ± 4 nm (magenta, D), 238 ± 7 nm (dark yellow, E), and 271 ± 2 nm (orange, F). TEM micrographs for **(B)** 46 nm PNDDS and **(C)** 271 nm PNDDS described in a dynamic light scattering (DLS) study (Zhang and Akbulut, 2011).

Zhang and Akbulut (2011) investigated the adsorption, desorption, and removal behavior of polymeric nanoparticulate drug delivery systems (PNDDS). Figure 2A shows the unimodal and relatively narrow intensity-weighted particle size distribution of PNDDS. It obtained six different particle size distributions of 46 ± 1, 81 ± 2, 159 ± 1, 197 ± 4, 238 ± 7, and 271 ± 2 nm. Based on TEM images, they observed core-shell spherical PNDDS (Figures 2B,C). The size had an effect on the adsorption, desorption, and removal of polymeric nanomedicine. Experimental results indicated that the removal rate of the particles increased with the increased particle size. More recently, a cross-linked cellulose aerogel from rice straw with a density of 2.2–24 mg cm^−3^ and porosity of 98.4–99.8% was achieved by a freeze-drying process (Dilamian and Noroozi, 2021). Different shapes of two types of dilute cellulose suspensions were found. The aerogels had a specific area of 178.8 m^2^ g^−1^ and mesopore volume of 0.8 cm^3^ g^−1^. It achieved adsorption capacities up to 170 g g^−1^ for the super-hydrophobic and oleophilic cellulose aerogels. A cellulose-rich modified rice husk was also reported for the removal of MB and Al^3+^ (You et al., 2021). It carried out the uptake for MB of 50.15 mg g^−1^ and the uptake for Al^3+^ of 2.87 mg g^−1^. Authors suggested this was due to the adsorption mechanisms of MB including pore filling, π-π interaction, and electrostatic attraction, as well as the adsorption mechanisms of Al^3+^, such as surface complexation, n-π interaction, and ion exchange. The functionalization of cellulose with hyperbranched polyamide was prepared for adsorption of orange II (OT) and Cu^2+^ ions (Yu et al., 2019). It achieved maximum sorption capacities of 976 mg g^−1^ for OT and 138 mg g^−1^ for Cu^2+^ ions. Authors indicated an electrostatic interaction for OT adsorption and the complexation/chelation of Cu^2+^ adsorption.

## Adsorbents Produced by Modification and Functionalization of Carboxymehyl Cellulose

Carboxymehyl cellulose (CMC) is the carboxymehyl group-substituted product of cellulose. CMC has the characteristics of high viscosity, adhesion, acid resistance, and physiological harm, which is widely used in food, medicine, papermaking, and textiles. According to the reports in the literature, it should be pointed out that the modification and functionalization of CMC are widely applied to remove heavy metals ions and organic pollutants. Bacterial cellulose (BC) was found to have important application potential (Ma et al., 2021; Mai et al., 2021; Wang X. et al., 2021; Guo et al., 2022). The early work had been done by Cavus et al. (2006). They developed a crosslinked hydroxyethyl cellulose-g-poly (acrylic acid) graft copolymer to remove Pb^2+^, Cu^2+^, and Cd^2+^. In Chen’s work, it was reported that carboxymethylated-BC performed better adsorption with values of 12.63 mg g^−1^ (copper) and 60.42 mg g^−1^ (lead) (Chen et al., 2009), compared with the values of 9.67 mg g^−1^ (copper) and 22.56 mg g^−1^ (lead) of BC. Obviously, carboxymethylated-BC had better adsorption performance. They demonstrated the pseudo-second-order kinetic model and the Langmuir model. Yang et al. (2011) applied CMC hydrogel beads with the maximum adsorption capacity of 6.49, 4.06, and 5.15 mmoL g^−1^ for Cu^2+^, Ni^2+^, and Pb^2+^, respectively. Ali (2012) discovered that the CMC and 2-acrylamido-2-methyl propane sulfonic acid hydrogels showed a great capability and were reused at least five times to recover toxic metal ions such as Mn^2+^, Co^2+^, Cu^2+^, and Fe^3+^ from their aqueous solutions. Undoubtedly, the modification and functionalization of CMC displayed amazing absorption capacity for various heavy metals ions. The cellulose acetate/sulfonated poly (ether ketone) blend ultrafiltration membranes were applied for the separation of Cr^3+^ ions from aqueous streams by ultrafiltration processes (Arthanareeswaran et al., 2007a). Dewangan et al. (2011) also developed the removal of Cr^6+^ by adsorption beads of sodium alginate and CMC.

**FIGURE 3:**
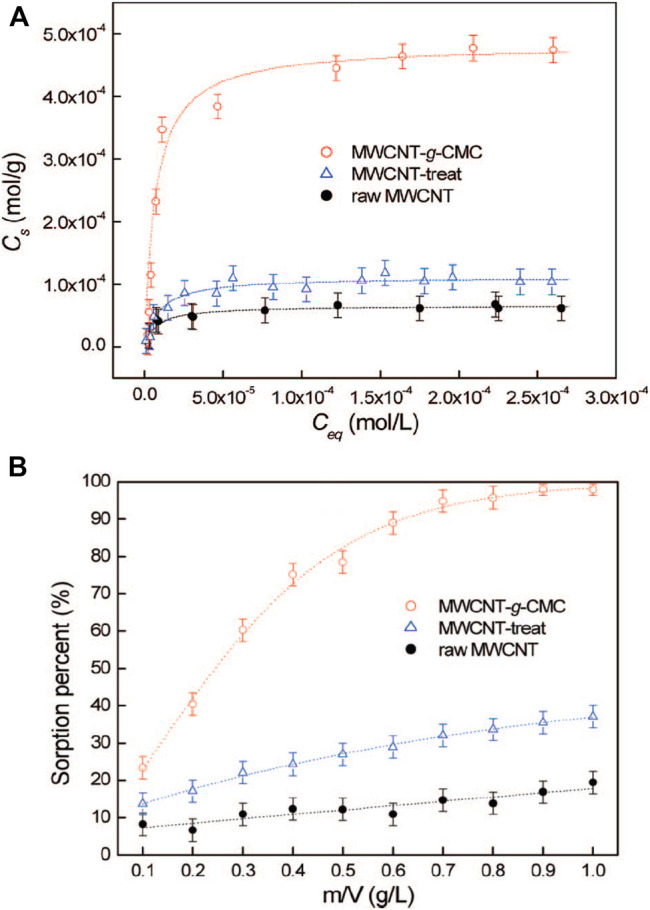
Sorption isotherms **(A)** and effect of sorbent content **(B)** on the removal of UO_2_^2+^ from solution on raw MWCNT, on MWCNT-treat, and on MWCNT-*g*-CMC. *T* = 25 ± 2°C, equilibrium time 24 h, pH = 5.0 ± 0.1, C [NaClO_4_] = 1.0 × 10^‒2^ mol/L **(A)** m/V = 0.4 g/L, **(B)** C [UO_2_^2+^]_(initial)_ = 2.00 × 10^‒4^ mol/L (Shao et al., 2009).

There are also some reports on the removal of individual metal ions. For example, the removal of arsenic ions was reported using a crosslinked sodium alginate/CMC adsorbent (Tiwari et al., 2008). Shao et al. (2009) developed the application of CMC grafted multiwalled carbon nanotubes (MWCNT-*g*-CMC) by using plasma techniques in the removal of UO_2_^2+^ from the aqueous solution, which had much higher sorption capacity than raw MWCNT. It obtained the sorption capacity of 6.0 × 10^–5^ mol/g for raw MWCNT, 1.1 × 10^–4^ mol/g for MWCNT-treat, and 4.7 × 10^–4^ mol/g for MWCNT-g-CMC (Figure 3A). MWCNT-g-CMC displayed a sorption percentage of UO_2_^2+^ from ∼23% to ∼98% with the increasing MWCNT-g-CMC content from 0.10 to 1.0 g/L (Figure 3B). Zhang et al. (2010) demonstrated that the carboxylate-functionalized cellulose possessed excellent adsorption capacity of 84.4% for Pb^2+^, which was significantly higher than that of unmodified cellulose. These examples further implied that modification and functionalization improved the adsorption performance. The ethylenediaminetetraacetic acid-functionalized magnetic Fe_3_O_4_ chitosan oligosaccharide and CMC nanocomposite adsorbent was fabricated for Pb^2+^ adsorption (Lian et al., 2020). The adsorption capacity for monolayer chemical adsorption was 432.34 mg g^−1^. The nanocomposite exhibited a Pb^2+^ removal rate of ∼100% using metal ion solutions of 100 and 200 mg L^−1^.

**FIGURE 4:**
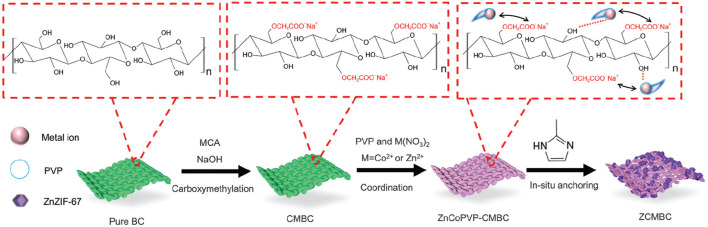
Schematic illustration of the synthesis of ZCMBC composite films (Mai et al., 2021).

**FIGURE 5:**
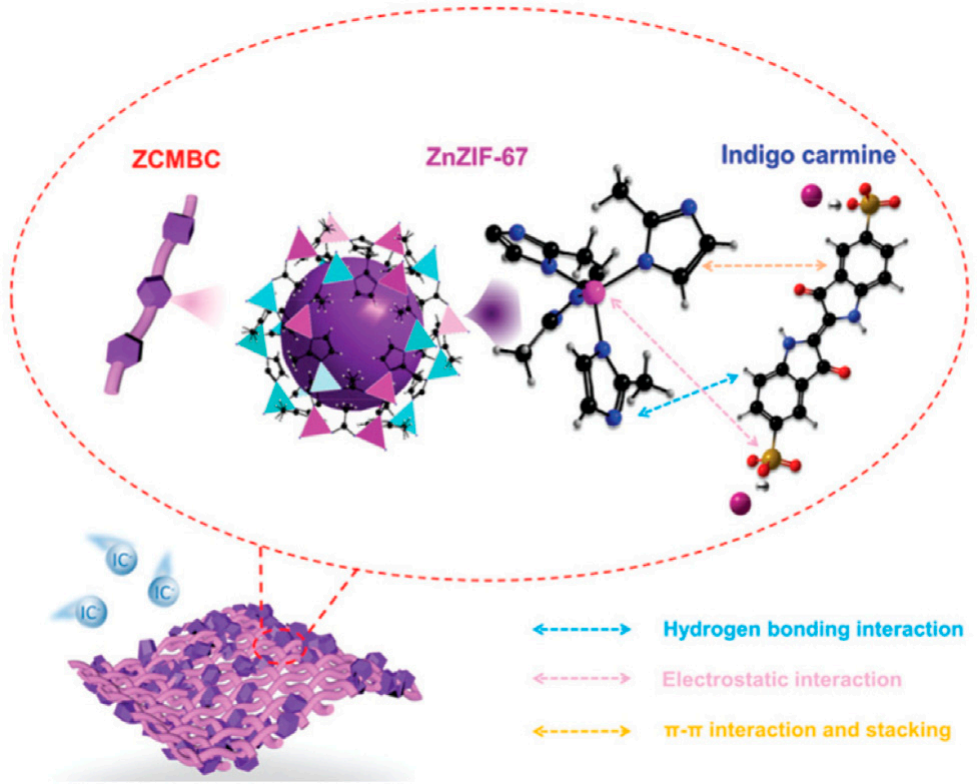
Proposed schematic diagram for adsorption mechanism of IC^−^ by ZCMBC composite films. The atoms are colored as follows: carbon (black), sulfur (earthy yellow), oxygen (red), zinc (light blue), cobalt (pink), sodium (wine red), hydrogen (grey), and nitrogen (dark blue). The light purple sphere indicates the cavity of ZnZIF-67 (Mai et al., 2021).

As mentioned above, it is very important to remove organic pollutants from wastewater. Ghanta et al. (2005) applied cellulose acetate blend and aromatic polyamide hydrazide reverse osmosis membranes for their separation behavior of phenol from phenol-water mixtures. Then, Eberhardt et al. (2006) reported the removal of (ortho) phosphate by refined aspen wood fiber treated with CMC and ferrous chloride. After that, De Smet’s group developed a super-flux cellulose triacetate dialyzer membrane to remove non-protein-bound and protein-bound uremic solutes (De Smet et al., 2007). The application of a microporous cationic hydrogel of hydroxypropyl cellulose in the removal of anionic dye was carried out by Yan et al. (2009). It was found that the adsorbent displayed an adsorption capacity of 2,478 g kg^−1^ for anionic dye AO7 at pH 3.96. Bodalo et al. (2005) reported the behavior of cellulose acetate membranes by reverse osmosis of ammonium aqueous solutions. The modification and functionalization of CMC also had the ability to remove protein. Metal ion separation and protein removal were also investigated using modified cellulose acetate membranes by Arthanareeswaran et al. (2007b). The millimeter-sized chitosan/CMC hollow capsules were prepared to remove three typical dyes such as MB, methyl orange (MO), and acid blue-113 (AB) by mixing and stirring positively charged chitosan and negatively charged CMC solutions under an electrostatic interaction (Kong et al., 2020). They achieved removal capacities of 64.6 mg g^−1^ for MB, 334.8 mg g^−1^ for MO, and 526.8 mg g^−1^ for AB. CMC/carboxylated graphene oxide composite microbeads were achieved to remove cationic MB dye (Eltaweil et al., 2020). They had an adsorption capacity of 180.32 mg g^−1^. The adsorbent had better reusability for nine cycles with improved adsorption properties. More recently, Mai et al. (2021) applied an *in situ* anchoring method to prepare zeolitic imidazolate frameworks (ZIFs)@carboxymethylated bacterial cellulose (ZCMBC) composite films (Figure 4). The ZCMBC composite films were reported to have a high ZIFs loading rate and satisfactory selective indigo carmine removal efficiency (98.7%). The authors suggested the adsorption mechanism of IC^−^ by ZCMBC composite films (Figure 5).

Millimeter hollow CMC microspheres/poly (ethyleneimine) (PEI) microspheres were fabricated for dye adsorption (Yang et al., 2021). The hollow microspheres had an adsorption capacity of 452 mg g^−1^ for MB. CMC and genipin crosslinked carboxyalkyl-chitosan combined with a sulfonated graphene oxide sponge with multiple active sites was fabricated for adsorbing sulfonamide antibiotics (Liu Y. et al., 2021). The composite showed high sulfamethoxazole and sulfapyridine. It obtained an adsorption capacity of 312.28 mg g^−1^ for sulfamethoxazole and 161.89 mg g^−1^ for sulfapyridine at 298 K. CTAB-modified CMC/bagasse cryogels were reported to remove bisphenol A (BPA), MB, and Cr^6+^ ions in binary or ternary aqueous mixtures (Meneses et al., 2022). They exhibited a removal rate of 100% for MB, 70% for Cr^6+^, and 95% for BPA. They observed an increased adsorption capacity of Cr^6+^ ions in the binary mixture or ternary mixture due to the synergistic effect.

## Adsorbents Produced by Cellulose-Based (Nano) Composites

It is known that (nano) composites are composed of two or more kinds of materials, which have synergistic effects and new properties, compared with an individual component. It means that cellulose-based (nano) composites were fabricated by using cellulose as the matrix and using inorganic or organic materials as reinforcement. In the literature, there are reports about the applications of cellulose-based (nano) composites. It is of great importance for cellulose-based (nano) composites adsorbents to remove heavy metals ions and organic pollutants. In early 2005, Guo and Chen (2005) completed pioneering work to remove heavy metals ions by using cellulose-based composites as adsorbents. They firstly applied cellulose/iron oxyhydroxide to remove arsenate and arsenite from groundwater. It was reported that the adsorbents displayed high removal efficiency of arsenite (99.6 mg g^−1^) and arsenate (33.2 mg g^−1^) at pH 7.0. They also summarized three reasons for and five advantages of cellulose/iron oxyhydroxide, such as cheap resources, recycled materials, excellent mechanical strength, high adsorption capacity, and high regeneration efficiency. Then, they used EXAFS to investigate the removal mechanism of cellulose/iron oxyhydroxide for arsenic (Guo et al., 2007). From then on, many groups explored the applications of cellulose-based (nano) composites in the removal of heavy metals ions and organic pollutants. For example, Maliyekkal et al. (2010) reported the applications for Pb^2+^ removal from aqueous solutions by using cellulose/manganese oxide nanocomposites. The physic-sorption played a dominant role in the adsorption of Pb^2+^. Nata et al. (2011) made amine-rich magnetite/BC nanocomposites by the solvothermal reaction. It was found that these nanocomposites displayed an adsorption capacity towards As^5+^ ions. Kumar et al. (2012) reported a cellulose-montmorillonite composite with an adsorption capacity of 22.2 mg g^−1^ for the detoxification of Cr^6+^ ions from industrial wastewater by a column methodology. It should be noted that the composite was reused for 10 adsorption-desorption cycles.

It is worth noting that hydroxyapatite has an excellent ion-exchangeability. In 2008, Choi and Jeong (2008) applied a hydroxyapatite/cellulose composite to remove heavy metals in aqueous solution. Islam et al. (2011) evaluated the feasibility of cellulose/carbonated hydroxyapatite nanocomposites for As^5+^ removal with the adsorption capacity of 12.72 mg g^−1^. Authors found the chemical process in the adsorption process. In the research of water treatment using adsorbents, it is very important to judge the chemisorption and/or physisorption process on the absorption mechanism. The above results indicated that cellulose/carbonated hydroxyapatite (nano) composites are promising adsorbents in the removal of heavy metals ions.

Similarly, cellulose-based (nano) composites including organic polymers are also applied in the removal of heavy metals ions. For example, Lima et al. (2005) developed chitosan/cellulose acetate film with an affinity for adsorbing copper. Highly porous adsorptive chitosan/cellulose acetate blend hollow fiber membranes were applied for copper ion removal in a batch adsorption mode (Liu and Bai, 2006). Cifci and Kaya (2010) used a poly (vinyl alcohol) (PVA)/cellulose membranes composite for metal removal from aqueous solutions.

There are a few reports about cellulose-based (nano) composites as adsorbents in the removal of organic pollutants. Cellulose acetate-supported Ni/Fe nanoparticles were first carried out to remove trichloroethylene from water by Wu and Ritchie (2006). Dridi-Dhaouadi et al. (2011) researched the sorption behavior of Pb^2+^ and C.I. acid yellow 44 on posidonia oceanica. Zhu et al. (2011) also applied magnetic cellulose/Fe_3_O_4_/activated carbon composites to remove CR. The nanocellulose hybrid containing polyhedral oligomeric silsesquioxane was also investigated to remove reactive dyes (Xie et al., 2011b). More recently, iron-based metal-organic framework@cellulose aerogels were obtained for CR dye adsorption by the *in situ* growing method (Huang et al., 2021a). It achieved an adsorption capacity of 280.3 mg g^−1^. Lemon peel/MCC hydrogels with porous structure and surface roughness were obtained for MB adsorption by direct co-dissolving in 1-butyl-3-methylimidazolium chloride (BmimCl) (Dai et al., 2021). The introduction of lemon peel increased the porosity and improved the thermal stability of the hydrogels. It obtained the maximum adsorption capacity of 57.54 mg g^−1^ for hydrogels.

**FIGURE 6:**
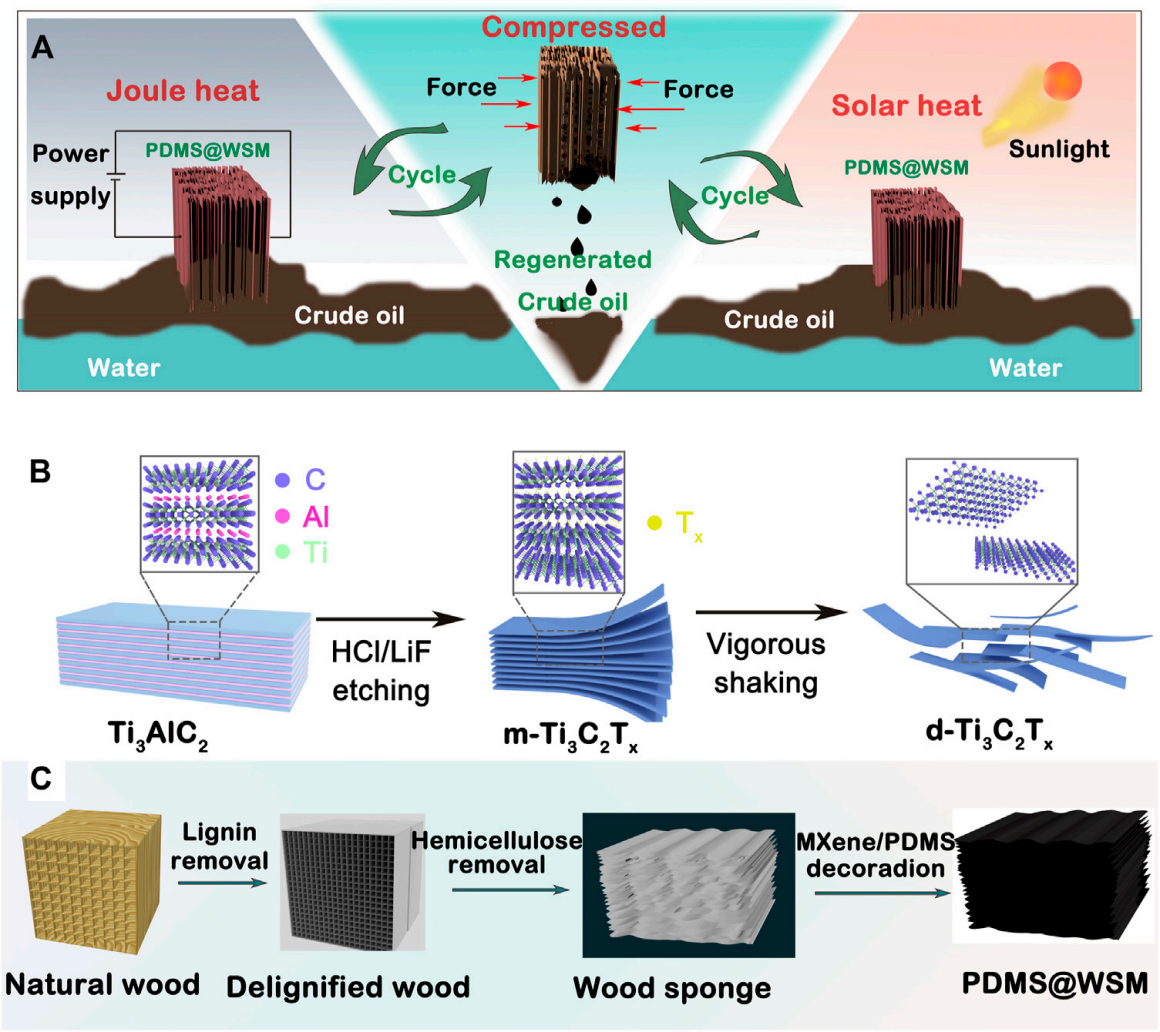
**(A)** Schematic illustration of Joule-heating and solar-heating PDMS@WSM to clean up and recover a viscous crude oil spill. **(B)** Schematic showing the synthesis process of the MXene nanosheet. **(C)** Schematic illustration of the fabrication of the wood sponge and PDMS@WSM (Wang et al., 2022).

The hollow cellulose/carbon nanotubes composite beads with aligned porous structure were fabricated by ice template and freeze-drying technology (Ding et al., 2021). All cellulose concentrations, pre-freeze temperatures, and voltages affected the hollow structure and diameter of the beads. Authors discovered the enhanced diameter of the beads with the increase of cellulose concentration, the different structure of beads at different pre-freeze temperatures, and the decreased diameter of beads with increased voltage. The composite showed good reusability, biodegradability, and an adsorption capacity of 285.71 mg g^−1^. A polyaniline/dicarboxyl acid cellulose@graphene oxide (GO) composite was synthesized to remove the reactive brilliant red K-2G (Liu T. et al., 2021). It obtained an adsorption capacity of 447.0 mg g^−1^ for the first scenario, and 729.0 mg g^−1^ during the subsequent photocatalysis process. A graphene oxide/cellulose nanocrystals nanocomposite was obtained to remove MB (Zaman et al., 2020). It removed around 98% of MB in 135 min and the maximum adsorption capacity was 751.88 mg g^−1^ Wang et al. (2022) also reported porous polydimethylsiloxane@wood sponge/MXene (PDMS@WSM) with outstanding compressibility and hydrophobic/lipophilic ability as a crude oil absorbent (Figure 6). The wood sponge consisted of cellulose by the removal of lignin and hemicellulose from natural wood. The PDMS@WSM had a maximum adsorption capacity of 11.2 × 10^5^ g m^−3^ due to the excellent Joule heating and photothermal conversion effect.

In fact, organic solvent can also improve the adsorption capacity of cellulose. Musyoka et al. (2011) reported ethylenediamine-modified cellulose with adsorption capacities of 0.0136 mmol g^−1^ for Cd^2+^ and 0.0179 mmol g^−1^ for Pb^2+^ ions. Xie et al. (2011a) synthesized nanocellulose hybrid biosorbents for adsorbing copper and nickel ions in aqueous solution. Hierarchical pore UiO-66/nanocellulose aerogels were prepared by a self-crosslinking method (Wang et al., 2019). The composite aerogels had adsorption capacities of 71.7 mg g^−1^ for anionic methyl orange and 51.8 mg g^−1^ for cationic MB. TEMPO-CNC MWCN/GO composite films were reported with a partition coefficient of 872.266 ml g^−1^ (Zheng et al., 2020). Porous cellulose/chitosan spheres were prepared to adsorb Cu^2+^ (Wittmar et al., 2020). They obtained a maximum adsorption capacity of 52.5 mg g^−1^ due to the amino group in the chitosan unit. Park et al. (2020) prepared poly (acryloyl hydrazide)-grafted CNC particles for adsorption of Cr^6+^ by strong electrostatic, hydrogen bonding, and chelating interactions. The adsorbents exhibited a high Cr^6+^ adsorption capacity of ∼457.6 mg g^−1^ by intra-particle diffusion resistance. CNC/iron oxide composites were prepared to remove arsenic (Dong F. et al., 2020). They obtained the maximal amount of 13.866 mg g^−1^ for As^3+^ and 15.712 mg g^−1^ for As^5+^. They exhibited chemical adsorption of monolayers. A TEMPO-oxidized CNF/magnetite was prepared to adsorb lead ions with a removal rate of 80% (Abou-Zeid et al., 2021). An MCC/magnesium sulfate hexahydrate (MCC/MH) composite adsorbent was used to adsorb heavy metal Co^2+^ ions (Wang R. et al., 2021). It obtained a removal rate of 97.67% and adsorption capacity of 153.84 mg g^−1^. Dialdehyde cellulose/GO composites adsorbents with high carboxyl groups density, high surface area, and low crystallinity index were obtained in both heterogeneous and homogeneous systems (Wang Z. et al., 2020). The adsorbents showed the adsorption capacities of 74.2 mg g^−1^ for Cu^2+^ and 91.7 mg g^−1^ for Pb^2+^. CNF/PVA composite gel spheres with 1–3 mm were prepared for organic pollutants and heavy metals ions (Yi et al., 2022). The spheres showed adsorption properties for simulated pollutants, including Cu^2+^, phenol, and aniline in water. They achieved a maximum absorption capacity of 17.22 As^3+^ and 15.712 mg g^−1^ for As^5+^ mmol g^−1^ for Cu^2+^, 176.72 mg g^−1^ for phenol, and 341.93 mg g^−1^ for aniline. The composite spheres also had good absorption properties for petroleum ether, ethyl acetate, and toluene. A liquid nitrogen directional freezing method was used to prepare CNF/chitosan/montmorillonite aerogels for wastewater treatment (Rong et al., 2021). The aerogels had a homogeneous three-dimensional directional pore structure, good mechanical properties, good adsorption performance, and reusability. They exhibited an adsorption capacity of 181.92 mg g^−1^ for Cu^2+^, 170.19 mg g^−1^ for Pb^2+^, and 163.85 mg g^−1^ for Cd^2+^.

## Mechanism of Adsorption

As mentioned above, it seems that the mechanism of adsorption can be simply divided into physisorption and chemisorption. In fact, it is believed that there exists simultaneous physisorption and chemisorption. In the adsorption process of adsorbents, the main mechanism still needs to be determined. As described in the literature, the physical forces include Van der Waals forces, hydrophobicity, hydrogen bonds, polarity and steric interaction, dipole induced dipole interaction, and pep interaction. Generally, it is reported that physisorption has the characteristics of low adsorption heat, does not require activation energy, single or multi molecular layer adsorption, no structure change of adsorbed molecules, no form of new chemical bonds, and is reversible. For the chemisorption process, it is considered that the adsorption heat is approximately equal to the reaction heat. The adsorption force is similar to the chemical bond and much stronger than Van der Waals forces. Chemisorption, as a single molecular layer adsorption, is selective and can be described by the Langmuir isotherm. In addition, chemisorption displays irreversibility to temperature and pressure, which requires activation energy. At present, the adsorption mechanisms are researched by adsorption kinetics, adsorption isotherms (Langmuir isotherm and Freundlich isotherm), and thermodynamics, which could explain the problems of physisorption and chemisorption, monolayer and multilayer adsorption. Therefore, it is very important to determine the type of adsorption. First, it can be judged according to the value of adsorption heat. The value of chemisorption heat is similar to that of the chemical reaction. In general, the value of chemisorption heat ranges from 83,740 to 418,680 J mol^−1^, while the value of physisorption heat is approximately 20,000 J mol^−1^. Secondly, the effect of temperature on the adsorption rate should be explored in the near future. During the chemisorption process, the adsorption rate is an activated process, which increases with the increase of temperature. However, physisorption is not an activated process, which has a high adsorption rate even at low temperatures. One can judge the type of adsorption by different adsorption processes at different temperatures. In the research of adsorption, adsorption capacities, regeneration efficiency, and selectivity are important factors. Moreover, in view of the above mentioned physisorption and chemisorption, one can conclude that chemisorption may have a high adsorption capacity and good selectivity due to chemical properties. However, physisorption has poor selectivity, which depends on the characteristics of the adsorbents.

Although most groups have investigated the mechanism of the adsorption process, we still have to say that it is not enough. For example, the famous Langmuir isotherm is based on the following assumptions of the uniformity of adsorbent surface, monolayer adsorption, dynamic adsorption, without force between the adsorbate molecules, etc., The Langmuir adsorption isotherm can be used in the low pressure range. When the gas pressure is higher in the adsorbate, close to the saturation vapor pressure, the equation produces a deviation due to the condensation of the adsorbate in the micro capillary and no single molecule layer adsorption. As for the Freundlich isotherm, it is an empirical formula in a narrow pressure range. In the low pressure or high pressure region, it cannot obtain satisfactory experimental results. Therefore, it is necessary to combine the new measurement method with molecular dynamics simulation to explore the adsorption mechanism. In fact, we believe that the adsorption is a very complex process, and some adsorption processes also include a chemical reaction and ion-exchange. Understanding the adsorption mechanism is of great significance for the synthesis and application of adsorbents.

## Future Perspectives

We believe that there is an increasing demand for environmentally friendly and economically friendly adsorbents in the field of water treatment. As discussed in this review article, cellulose is one of the polysaccharides composed of glucose molecules and the most abundant natural renewable biomass in the world. Undoubtedly, the modification and functionalization of cellulose and cellulose-based (nano) composites meet these requirements. Although there is a long road ahead for these applications, cellulose has a very bright future as an amazing and promising bio-adsorbent for wastewater treatment. More importantly, we expect that low-cost and greener bio-adsorbents will open a new window for the high value-added applications of cellulose, compared with other adsorbents. Besides heavy metals ions and organic pollutants, the modification and functionalization of cellulose and cellulose-based (nano) composites were also reported for protein adsorption (Zhao et al., 2021), drugs adsorption and release (Zhang et al., 2019), bilirubin (Wang Y. et al., 2021), carbon dioxide (CO_2_) adsorption (Sepahvand et al., 2020), and lysozymes (Rahmatika et al., 2020).

As described by Ali et al. (2012), there are many issues that need to be solved in the next step, such as adsorption mechanism, industrial scales preparation, regeneration, specific surface area, the management of removed pollutants, dispersion, etc., We would like to point out that there are many requirements for adsorbents in wastewater treatment, such as inexpensive, eco-friendly, environmentally friendly, good selectivity, high adsorption capacities, good regeneration efficiency, broad spectrum, etc., As we all know, it is difficult to obtain perfect adsorbents. Therefore, finding a balance among all factors is very important for practical applications.

The adsorbents are expected to have practical applications, which is the first and only standard to measure the quality of adsorbents. It is necessary to investigate the mechanism and eliminate interference at lab-scale batch studies. Obviously, unlike lab-scale batch studies, industrial waste water containing various heavy metals ions and organic pollutants is more complex. Sometimes, it was found that the adsorbents displayed good performance at lab studies and poor performance in industrial wastewater. At least, the absorbent with good performance at lab studies should be characterized and tested with industrial wastewater. So, the design of adsorbents should be based on realistic industrial applications, not the opposite. As described above, hundreds of cellulose-based bio-adsorbents were reported in the literature. However, few types of bio-adsorbents can be used in practical applications. The modification and functionalization of cellulose, CMC, and cellulose-based (nano) composites could be used to create bio-adsorbents to remove heavy metals ions and organic pollutants. The future development direction is put forward from the aspects of adsorption mechanism, theoretical simulation, and experimental verification. Although there is no common standard to judge these bio-adsorbents, it is necessary to narrow the scope.

In addition, it is worth noting that the main components of biomass are polysaccharides (cellulose and hemicellulose) and lignin. Cellulose is obtained by pretreatment of biomass. It seems that the development of pretreatment methods on biomass is of great importance to broaden its industrial applications. Obviously, the pretreatment methods determine whether cellulose and cellulose-based (nano) composites are economically friendly adsorbents. In general, cellulose can be used as a raw material to produce bio-based fuels, chemicals, and materials. Therefore, cellulose-based bio-adsorbents should have high value-added and low price capabilities, compared with other applications such as bio-based fuels, chemicals, and materials. Of course, the high regeneration efficiency of bio-adsorbents would contribute to reduce the cost.

Moreover, science and technology are like brothers. The problems of the adsorption mechanism, preparation, property, etc., may belong to the scientific field. However, the application in the wastewater field may belong to the technical field. It is found that the problems of technology always determine the quality and price of bio-adsorbents in the industrial process. So, there is still a long way to go from lab-scale batch studies to large-scale industrial applications of bio-adsorbents. We would like to point out that the research in the lab should meet industrial applications and resolve industrial problems, not the opposite.

## Conclusion

In this review article, we summarized the recent progress of adsorbents produced by modification and functionalization of cellulose and cellulose-based (nano) composites to remove heavy metals ions and organic pollutants. We believe that the modification and functionalization of cellulose, carboxymehyl cellulose, and cellulose-based (nano) composites are amazing and promising methods to create bio-adsorbents in the field of water treatment. It is expected that cellulose and cellulose-based (nano) composites will have promising applications in the field of wastewater treatment.
